# The dual impact of antiretroviral therapy and sexual behaviour changes on HIV epidemiologic trends in Uganda: a modelling study

**DOI:** 10.1136/sextrans-2013-051219

**Published:** 2014-02-24

**Authors:** Leigh Anne Shafer, Rebecca N Nsubuga, Ruth Chapman, Katie O'Brien, Billy N Mayanja, Richard G White

**Affiliations:** 1 Department of Internal Medicine, University of Manitoba, Winnipeg, Manitoba, Canada; 2 Medical Research Council Unit on AIDS/Uganda Virus Research Institute, Entebbe, Uganda; 3 London School of Hygiene and Tropical Medicine, London, UK

**Keywords:** Antiretroviral therapy, HIV/AIDS, sexual behavior, epidemiologic trends, mathematical modeling, Africa, Uganda

## Abstract

**Objectives:**

Antiretroviral therapy (ART) availability in a population may influence risky sexual behaviour. We examine the potential impact of ART on the HIV epidemic, incorporating evidence for the impact that ART may have on risky sexual behaviour.

**Methods:**

A mathematical model, parameterised using site-specific data from Uganda and worldwide literature review, was used to examine the likely impact of ART on HIV epidemiologic trends. We varied assumptions about rates of initiating ART, and changes in sexual partner turnover rates.

**Results:**

Modelling suggests that ART will reduce HIV incidence over 20 years, and increase prevalence. Even in the optimistic scenario of ART enrollment beginning after just five months of infection (in HIV stage 2), prevalence is estimated to rise from a baseline of 10.5% and 8.3% among women and men, respectively, to at least 12.1% and 10.2%, respectively. It will rise further if sexual disinhibition occurs or infectiousness while on ART is slightly higher (2% female to male, rather than 0.5%). The conditions required for ART to reduce prevalence over this period are likely too extreme to be achievable. For example, if ART enrolment begins in HIV stage 1 (within the first 5 months of infection), and if risky sexual behaviour does not increase, then 3 of our 11 top fitting results estimate a potential drop in HIV prevalence by 2025. If sexual risk taking rises, it will have a large additional impact on expected HIV prevalence. Prevalence will rise despite incidence falling, because ART extends life expectancy.

**Conclusions:**

HIV prevalence will rise. Even small increases in partner turnover rates will lead to an additional substantial increase in HIV prevalence. Policy makers are urged to continue HIV prevention activities, including promoting sex education, and to be prepared for a higher than previously suggested number of HIV infected people in need of treatment.

## Introduction

Antiretroviral therapy (ART) use is now widespread in sub-Saharan Africa.^1^ Recent evidence suggests that ART may reduce population-level incidence of HIV.^2^ Modelling studies have also investigated the potential impact of ART.^3–12^ These studies have reached varying conclusions. Treatment prolongs the lives of those infected, so some studies have suggested increased prevalence due to ART.^6^ ^9^ ^13^ However, it has also been suggested that frequent testing and early ART enrolment could reduce HIV prevalence,^7^ ^8^ ^10^ and may even eradicate the epidemic.^10^ ^14^ HIV-infected individuals on ART are less infectious than those not on ART.^15–17^ The differing conclusions are largely dependent on assumptions, and may be particularly sensitive to assumptions about sexual behaviour.^12^ ART may lead to sexual disinhibition as people feel that they may live a long life with HIV.

The overall impact that ART may have on the HIV epidemic sometimes incorporates the potential impact of ART on sexual behaviour.^6^ ^10^ However, the ranges of potential behaviour change are often not based on data, and do not include the impact that ART availability may have on behaviour among HIV uninfected people. Previous studies have provided contrasting results regarding the impact of ART on sexual behaviour.^18–20^

Previously, we examined self-reported evidence for changing sexual behaviour after the introduction of ART in a rural Ugandan cohort in 2004.^21^ We found evidence that risky behaviour, particularly partner turnover rates, may rise among HIV uninfected people in response to the availability of ART.

In the Ugandan cohort mentioned above, HIV prevalence rose from 6.87% in 2004, the year that ART roll-out began, to 8.75% by 2012. However, in this cohort, most people do not begin ART treatment until HIV stage 3.

Here, through mathematical modelling, we assess the plausible impact of ART on future HIV prevalence and incidence under different scenarios of rate of ART enrolment. As some have postulated that ART may be a means of eradicating HIV,^10^ ^14^ our objective is to assess whether ART could conceivably reduce or eliminate the HIV epidemic, under extremely optimistic conditions. We examine impact in a range of scenarios with varying assumptions about the average time from HIV infection until ART enrolment, infectiousness while on ART, and sexual behaviour modification. Uniquely in this study, we incorporated evidence-driven potential sexual behaviour change due to ART among people infected with HIV, as well as due to the availability of ART among those uninfected with HIV.

## Methods

In 1989, the Medical Research Council established a general population cohort (GPC) in rural Uganda.^22–24^ Participants are serotested annually for HIV. We present HIV prevalence estimates from this cohort, following estimation methods described previously.^25^ ^26^ We fit our model to prevalence data through 2004, which is the last year in which the impact of ART on epidemiologic trends would not yet have been felt in this population.

### Mathematical model

**Figure 1:**
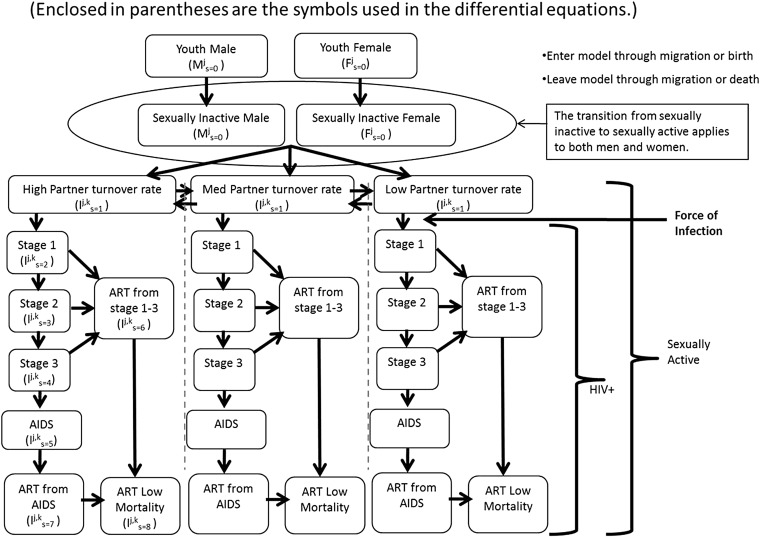
Compartmental Mathematical Model (Enclosed in parentheses are the symbols used in the differential equations.)

The compartmental mathematical model is stratified by age, sex and sexual activity group (figure 1). Details are online (see online supplementary appendix). Results are based on simulations with average durations in HIV stages 1–4 of 5 months, 7 years, 18 months and 10 months.

Upon ART enrolment in the model, infectiousness drops. At a rate of 0.5/year, those who have not yet died move into a compartment with mortality rate equivalent to background mortality. It is assumed that after surviving the initial ART period, life expectancy on ART is the same as that among HIV uninfected people.

### Baseline scenario

The baseline scenario was fit to data from Uganda and literature reviews. We fit to empirically estimated HIV prevalence by gender among 15–54-year-olds from 1991 to 2004, before ART can have impacted prevalence. To fit, we ran 750 000 simulations with varying parameter values (see online supplementary table S1). Parameters include behaviour change before the introduction of ART, consistent with evidence suggesting that risky behaviour declined during the 1990s.^27^ The parameter set with the highest goodness of fit between model and empirically estimated prevalence was considered the best fitting.

### ART impact scenarios

ART introduction was simulated in 2004. We examined six ART scenarios (table 1).

**Table 1:** ART Impact Scenarios** **

Scenario	Probability of HIV transmission while on ART (per partnership)	Earliest HIV stage of ART enrolment	Modelled change in sexual partner turnover?
1	0.5% F->M, 1.0% M->F	3 (rate of initiation varies)	No
2	2.0% F->M, 4.0% M->F	3 (rate of initiation varies)	No
3	0.5% F->M, 1.0% M->F	1 (rate of initiation varies)	No
4	2.0% F->M, 4.0% M->F	1 (rate of initiation varies)	No
5	0.5% F->M, 1.0% M->F	2 (rate of initiation=0.9/year)	Yes
6	2.0% F->M, 4.0% M->F	2 (rate of initiation=0.9/year)	Yes

ART, antiretroviral therapy.

Within each of the first four scenarios, we varied the annual rate of ART enrolment once the respective HIV stage was reached, from 0.0 (baseline—no ART) to 1.0. In these scenarios, we assumed that partnership turnover rates did not change after ART introduction. We examined two scenarios in which ART enrolment may begin as early as HIV stage 1, and two in which ART enrolment may begin in HIV stage 3. We present the scenarios of ART enrolment beginning in stages 1 and 3 as these are boundaries. It may be unlikely to expect ART enrolment in stage 1 often enough to impact the epidemic, but we present this scenario as it represents the upper bound of the impact that ART could possibly have on the HIV epidemic.

When examining the impact of increasing partner turnover rates in response to ART availability, it was assumed that ART enrolment may begin as early as HIV stage 2, and that the enrolment rate was 0.9/year. The percent increase in partner turnover rate beginning in 2004 varied from 0.0% (no change) to 50.0%. HIV stage 2 represents a reasonable yet aggressive timing for ART enrolment.

When HIV transmission while on ART was 1% male to female (0.5% female to male), this represented a percent reduction in transmission probability in our best fitting scenario among men ranging from 85.1% to 96.6%, and among women ranging from 90.9% to 97.6%. The percent reduction depended on the stage from which ART was initiated, and the probability of transmission from the respective stage before ART enrolment.

### Goodness-of-fit measures and sensitivity analysis

We assessed the sensitivity of results to parameter value set selection by summarising the results from multiple good fitting parameter sets: We found the best fitting parameter values using three goodness-of-fit (GoF) measures: a sum of squares, a χ^2^,^28^ and maximum likelihood.^29^ ^30^ We used three GoF measures because some readers may be more confident in results of one or another of the measures, but the three measures produced significantly overlapping best fits.^2^ We compared results using the first through seventh best fitting parameter sets.

Of the 21 sets of parameter values, 10 were repeats. That is, five of the seven best fitting sets found when maximising the likelihood function were the same as those found when minimising the sum of squared differences, and five of those found under the χ^2^ GoF had also been found under either the sum of squares or maximum likelihood GoF. We used the remaining 11 parameter sets to assess robustness of model results.

## Results

In the best fit, HIV transmission probability per partnership and stage is shown in online supplementary table S1.

### Prevalence

**Figure 2:**
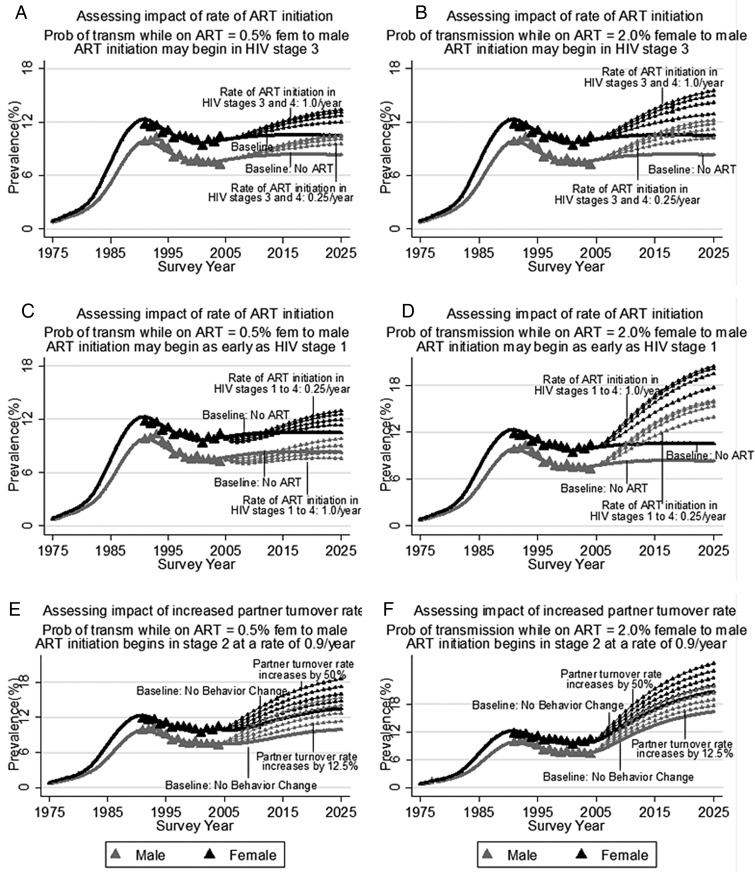
Sensitivity Analyses of Dual Impact of antiretroviral therapy (ART) and Potential Behavior Change on HIV Prevalence.

Among the 11 best fitting simulations, we obtained model-estimated prevalence continuously from 1975 to 2025. The mean of these model estimates is presented in figure 2, for a range of different scenarios. Panels A and B correspond to scenario sets 1 and 2, described in methods. With no ART, HIV prevalence reaches 10.5% among women and 8.3% among men by 2025. In panel A, if the rate of ART enrolment beginning in stage 3 is 0.25/year, then predicted HIV prevalence rises to 12.0% among women and 9.5% among men by 2025. Panel B portrays a scenario set in which the drop in the probability of HIV transmission while on ART is less than that in panel A. In this case, under an otherwise similar scenario to that just described, prevalence rises to 12.9% among women and 10.2% among men by 2025. The increase in HIV prevalence is more pronounced in these scenarios if people begin ART at a faster rate. If ART enrolment beginning in HIV stage 3 is 1.0/year, then HIV prevalence may rise to 15.5% among women and 12.2% among men by 2025 (panel B).

In scenario set 3, people initiate ART in HIV stage 1, and there is no behaviour change in response to ART availability. This is the only one among the range of scenarios examined in which the average of our 11 best fitting prevalence estimates indicates that HIV prevalence may initially fall after ART introduction (figure 2C). By 10 years after ART is introduced, among men, HIV prevalence is higher if the rate of ART enrolment is less than 0.50/year than it would have been in the absence of ART, and among women it is higher regardless of ART enrolment rates. However, among men, if the rate of ART enrolment is greater than 0.50/year, and ART enrolment begins in HIV stage 1, then HIV prevalence may permanently decline, relative to what it is in the absence of ART. If the rate of ART enrolment is 1.0/year, HIV prevalence falls to 10.5% among women and 7.4% among men by 2015, before rising to 11.4% among women by 2025 and remaining level among men. Panel D (scenario set 4) portrays a similar scenario set, except that the drop in probability of HIV transmission while on ART is less. In this case, the impact of ART is to increase HIV prevalence dramatically, regardless of the rate at which people initiate ART.

We next examined the impact that ART may have on HIV prevalence if partner turnover rates increase. The scenarios depicted in figure 2E,F, are described as scenario sets 5 and 6 in methods. In panel E, a 12.5% increase in partner turnover rate results in rising HIV prevalence up to 20 years after ART is introduced. By 2025, prevalence reaches 17.7% among women and 13.9% among men, contrasting with 12.1% (women) and 10.2% (men) without behaviour change. If ART only reduces HIV transmission probability per partnership to 2% female to male (panel F), then prevalence is higher, but the relative impact of higher partner turnover rates is similar to what it is under the scenario in which ART reduces HIV transmission probability to 0.5% female to male.

As anticipated, we estimated increasing HIV prevalence in the presence of ART even when most of the HIV infected are on ART. In figure 2EF, the rate of initiating ART was 0.9/year, beginning in stage 2. In this case, the percent of HIV infected who are on ART reaches almost 90% by 20 years after the introduction of ART in the population.

### Sensitivity analysis

**Figure 3:**
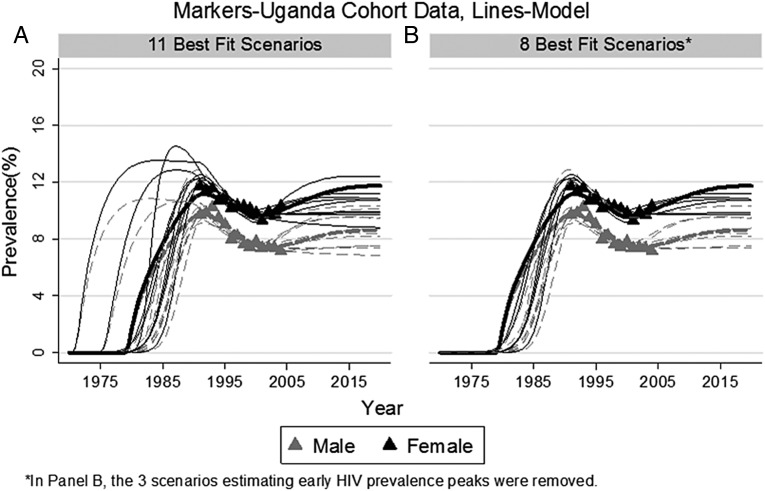
Prevalence by Gender–Age 15–54 Markers-Uganda Cohort Data, Lines-Model.

Multiple sets of parameter values produced model estimates that fitted the empirically estimated prevalence. Without ART, the 11 sets of parameter values that most closely fit our data estimated similar HIV epidemics (figure 3).

By 2025, all 11 sets of parameter values estimated that ART would result in increased HIV prevalence, if ART enrolment begins in HIV stage 3 (table 2, scenario 1). The smallest increase in prevalence would occur if the probability of HIV transmission per partnership female to male was low at 0.5%, and the rate of ART enrolment was also low at 0.25/year. In this case, by 2025, the mean increase in prevalence was 14.6% (table 2, scenario 1). If either the rate at which people begin ART treatment or the probability of HIV transmission while on treatment rises, then all 11 parameter sets estimated an even greater rise in HIV prevalence than under the scenario just described.

**Table 2:** Percent increase in HIV prevalence forecasted by 11 ‘best fit’ parameter value sets, under scenarios depicted in figure 2 (% increase compared to scenario of no ART or no behaviour change)

		Modelled parameter value set											
Scenario	Year	1	2	3*	4	5	6	7	8	9	10*	11*	Mean (SD)
Probability of transmission on ART=0.5% female to male. No ART enrolment in HIV stages 1 or 2 (figure 2A)													
Rate of ART init starting stage 3													
0.25/year	2015	1.3	11.2	0.4	−0.4	7.4	6.0	13.0	10.2	10.6	9.3	12.7	7.4 (4.9)
	2025	6.5	19.3	5.5	4.5	14.3	14.2	20.7	19.4	18.4	16.9	21.4	14.6 (6.3)
1.00/year	2015	1.8	23.4	−0.2	−3.7	14.4	11.5	28.6	21.8	23.2	20.2	27.9	15.4 (11.5)
	2025	9.9	39.0	7.9	1.6	26.8	27.2	43.7	39.5	38.2	33.9	45.0	28.4 (15.4)
Probability of transmission on ART=2.0% female to male. No ART enrolment in HIV stages 1 or 2 (figure 2B)													
Rate of ART init starting stage 3													
0.25/year	2015	6.6	14.8	4.9	2.5	15.5	14.8	17.8	17.6	16.2	16.8	15.0	13.0 (5.5)
	2025	16.7	24.1	13.9	10.4	25.8	27.1	27.0	31.5	26.2	31.2	24.7	23.5 (6.9)
1.00/year	2015	14.2	32.6	10.6	3.3	34.7	32.8	39.7	39.1	36.1	36.8	33.6	28.5 (12.7)
	2025	32.9	50.9	27.5	15.5	55.5	57.1	58.8	66.2	56.4	64.8	53.2	49.0 (16.3)
Probability of transmission on ART=0.5% female to male. ART enrolment begins in HIV stage 1 (figure 2C)													
Rate of ART init													
0.25/year	2015	−10.7	21.9	−10.7	−19.0	13.4	14.0	21.1	15.6	21.8	2.7	23.3	8.5 (15.4)
	2025	−4.1	44.7	−4.3	−21.5	30.1	37.5	42.3	39.4	44.2	14.4	49.2	24.7 (24.5)
1.00/year	2015	−30.9	17.1	−25.2	−33.6	6.7	10.9	17.2	7.7	24.5	−3.9	18.8	0.8 (21.2)
	2025	−41.1	35.5	−25.5	−46.8	12.0	27.4	32.2	21.6	47.5	1.2	43.7	9.8 (33.6)
Probability of transmission on ART=2.0% fem ale to male. ART enrolment begins in HIV stage 1 (figure 2D)													
Rate of ART init													
0.25/year	2015	16.0	44.2	12.2	−4.5	61.0	58.8	50.4	56.0	53.3	36.0	39.7	38.4 (21.6)
	2025	42.7	72.8	36.5	6.2	98.5	102.0	81.2	98.7	87.9	74.1	71.0	70.1 (30.1)
1.00/year	2015	17.2	69.9	14.7	−12.9	102.2	96.6	80.0	89.3	85.7	52.2	61.3	59.7 (37.9)
	2025	41.2	105.0	41.1	−10.6	151.5	153.9	119.2	143.1	129.8	95.5	100.4	97.3 (52.7)
Probability of transmission on ART=0.5% female to male. ART enrolment begins in stage 2 at rate 0.9/year (figure 2E)													
Change partner turnover rate													
Increase by 12.5%	2015	10.3	6.4	7.1	9.6	10.9	8.8	7.5	8.5	6.2	6.6	6.0	8.0 (1.7)
	2025	14.5	6.3	10.0	16.2	14.7	10.4	8.0	9.9	6.5	9.2	5.5	10.1 (3.6)
Increase by 50%	2015	43.2	19.9	32.9	65.6	40.9	34.4	25.0	31.0	24.0	30.9	20.6	33.5 (13.1)
	2025	58.7	19.2	45.7	110.0	48.0	36.1	25.4	33.9	24.1	43.2	18.6	42.1 (25.9)
Probability of transmission on ART=2.0% female to male. ART enrolment begins in stage 2 at rate 0.9/year (figure 2F)													
Change partner turnover rate													
Increase by 12.5%	2015	9.8	3.1	8.0	12.0	6.1	6.0	3.7	5.2	5.0	8.4	3.5	6.4 (2.8)
	2025	12.0	2.9	9.5	18.7	6.0	6.2	3.9	5.1	4.9	10.5	3.6	7.6 (4.7)
Increase by 50%	2015	41.6	11.6	35.1	71.1	24.8	24.8	14.4	20.6	20.7	38.7	12.9	28.8 (17.4)
	2025	47.8	10.8	39.2	101.2	23.4	25.1	15.3	20.2	19.4	47.8	13.1	33.0 (26.1)

*Though parameter value sets 3, 10, and 11 provided good model fits to empirically estimated prevalence, we believe they are less likely than the others because they estimated the HIV epidemic peaking too early (see figure 3).

ART, antiretroviral therapy.

In the extreme scenario in which ART was initiated beginning during HIV stage 1, HIV transmission probability on ART female to male was 0.5%, and there was no behaviour change, the estimated impacts from the 11 parameter sets were inconsistent. Three of the sets suggested a drop in HIV prevalence by 2025, while eight suggested an increase. Across all parameter sets, the mean change in HIV prevalence over the scenario of no ART was a 24.7% rise assuming that the rate of ART enrolment was 0.25/year and a 9.8% rise if the rate of ART enrolment was 1.00/year (table 2, scenario 3). If the probability of transmission while on ART was 2% female to male, all but one of the modelled parameter sets indicated that ART would result in rising HIV prevalence (table 2, scenario 4).

Not surprisingly, all sets of parameter values suggested that an increase in partner turnover rate would result in rising HIV prevalence. For example, if the probability of HIV transmission while on ART was just 0.5% female to male, and ART enrolment began in stage 2 at a very high rate of 0.9/year, then the mean increase in prevalence by 2025 caused by a 12.5% increase in partner turnover rate was 10.1% (table 2, scenario 5).

### Incidence

**Figure 4:**
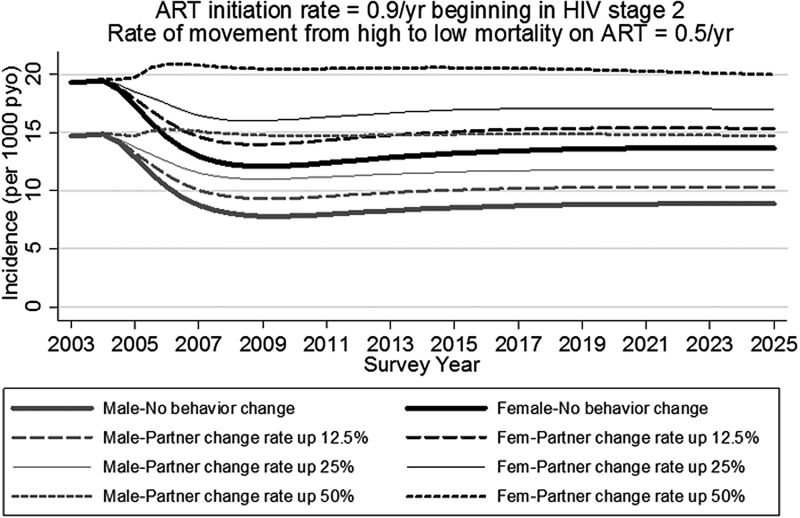
Incidence among age 15–54 antiretroviral therapy (ART) initiation rate=0.9/year beginning in HIV stage 2. Rate of movement from high to low mortality on ART=0.5/year.

Without behaviour change, ART reduces HIV incidence (figure 4). This assumes a high rate of ART enrolment beginning in HIV stage 2, and that ART lowers the probability of HIV infection per partnership to 0.5% female to male and 1% male to female. Without behaviour change, our model suggests that ART would lower incidence from 14.7 to 8.9 per 1000 person-years observation (pyo) among men, and from 19.3 to 13.7 among women. If average partner turnover rate increases by 12.5%, then our results indicate that the dual impact of ART and behaviour change would lower incidence to 10.3 per 1000 pyo among men, and to 15.3 among women. If partner turnover rate increases by 50%, then incidence would initially rise, but would then stabilise at approximately the same values as without ART.

In the figure 4 scenario, with no behaviour change, 18.4% of incidence by 2025 comes from people in stage 1, 6.6% from people in stage 2, and 73.4% from people on ART. Just 1.6% of incidence comes from people in stages 3 and 4. By contrast, in the same scenario but in the year prior to ART introduction, 36.2% of incidence comes from people in stage 1, 40.3% from stage 2, 16.5% from stage 3 and 7.0% from stage 4. The lower percent of incidence in stage 4 despite high infectiousness results from: a reduction in partner turnover rate in stage 4 (see online supplementary table S1), and mortality before stage 4 such that some people never reach this stage.

## Discussion

### Epidemiologic forecast

Recent evidence from clinical trials have shown the positive impact that ART may have on reducing new infections (incidence).^2^ Our modelling work supports this evidence by also showing a reduction in incidence, unless partner turnover rates increase greatly (>50%). However, although incidence falls, results suggested that HIV prevalence will be higher with ART than without. Among the scenarios examined, HIV prevalence may decrease among men only in an extreme case which may not be achievable in practice. That is, ART enrolment would need to begin while still in HIV stage 1, the rate of ART enrolment would need to be greater than 0.50/year, sexual partner turnover would not change, and the probability of transmission while on ART would be just 0.5% per partnership from female to male (representing >95% reduction in transmission compared to no ART). Even in this case, 8 of the 11 sets of parameter values that we assessed suggested that HIV prevalence would be higher in the presence of ART than in its absence.

Despite the short duration in the initial HIV stage of infection, our model estimates that in the absence of ART about 36% of incidence comes from partnerships formed in which the HIV-infected partner was in the highly infectious stage 1. Initiating ART during this stage, if feasible, would therefore have the greatest impact on the epidemic.

Previous studies have suggested a range of potential outcomes to rolling out ART, from increasing^6^ ^9^ ^13^ to decreasing HIV prevalence.^7^ ^8^ ^10^ ^14^ Although our work supports this range of potential outcomes to rolling out ART, in all but the most extreme scenarios our results consistently suggest that HIV prevalence will rise as a result of ART.

Results from this study come from a model that was parameterised and fit using data from Uganda. We expect that our results provide a good estimate of the impact of ART and corresponding sexual behaviour change on HIV epidemiologic trends in countries that are similar to Uganda in terms of HIV prevalence trends pre-ART and HIV modes of transmission (mainly heterosexual sex).

### Limitations

Results of any modelling study may be influenced by model assumptions. We have described our model assumptions in detail in the online supplementary appendix. One model assumption was that of no HIV/AIDS-related mortality after the initial period on ART. This is likely overly optimistic as non-perfect ART adherence speeds HIV progression and a higher mortality rate. If we had modelled a slightly higher mortality rate, even among those on ART, then estimated HIV prevalence after ART is introduced would be lower, as infected people would die at a higher rate. Also, we used a deterministic mathematical model. As such, stochastic events that may influence the plausible range of the impact of ART on HIV prevalence and incidence were not modelled.

In our best fitting model simulation, 36.2% of HIV incidence prior to ART introduction came from people in HIV stage 1. While this is similar to that estimated elsewhere,^31^ ^32^ it is higher than that estimated by some.^33^ If more HIV incidence is attributable to higher HIV stages than we estimated, then ART enrolment in later stages would have a greater impact on incidence and subsequent prevalence.

## Conclusion

It is unlikely that most HIV infections could be identified during the high viraemic stage 1 of infection. If infections are identified at a rate of 0.9/year beginning in stage 2, and ART is initiated immediately after detection, our results indicate that HIV incidence would fall as a result of ART. However, this comes at a cost. Our results still indicate rising prevalence as those infected live longer. The combined effect of increased risky sexual behaviour and longer life expectancy while infected would increase HIV prevalence dramatically. Care providers should be prepared for a higher than previously suggested^11^ number of people who are likely to need treatment.

**Key messages**

- Although HIV incidence will fall, HIV prevalence will rise as a result of antiretroviral therapy.
- If the possible increase in risky sexual behaviour is not controlled, HIV prevalence will rise even further.
- Policy makers must prepare to provide care for the increased population living with HIV.
- Policy makers are urged to continue HIV prevention activities, including promoting sex education.

## Supplementary Material

Web appendix

Web table
