# Supplementary material for: The dual impact of antiretroviral therapy and sexual behaviour changes on HIV epidemiologic trends in Uganda: a modelling study
Source: Sex Transm Infect. 2014 Feb 24;90(5):423–9. doi: 10.1136/sextrans-2013-051219 (PMC4112492; doi:10.1136/sextrans-2013-051219)
Supplement: Web appendix [file sextrans-2013-051219-s1.pdf]

Appendix I:
HIV Evolution Model
Description and Differential Equations

December, 2013

Contents

1 Model Description **3**
- 1.1 Burn In . . . 3
- 1.2 Age Group Parameters . . . 3
- 1.3 Sexual Activity Group Parameters . . . 3
- 1.4 Age/Sexual Activity Input Parameters . . . 3
  - 1.4.1 Birth rate . . . 3
  - 1.4.2 Background Mortality rate . . . 4
  - 1.4.3 Age of sexual debut and becoming sexually active . . . 4
  - 1.4.4 Desired partnership turnover rate . . . 4
  - 1.4.5 Desired partner mixing parameters . . . 4
  - 1.4.6 Cross generational mixing . . . 5
  - 1.4.7 Sexual Activity migration . . . 5
- 1.5 Population States . . . 5
  - 1.5.1 HIV state . . . 5
- 1.6 HIV State Input Parameters . . . 6
  - 1.6.1 HIV stage and ART transition rates . . . 6
  - 1.6.2 HIV/AIDS-related Mortality rate . . . 6
  - 1.6.3 HIV infection rate (force of infection) . . . 6

2 Model Equations **8**
- 2.1 Line 1 - Aging, Births and Deaths . . . 8
- 2.2 Line 2 - Sexual Activity Group Migration . . . 8
- 2.3 Line 3 - Sexual Activity Debut . . . 9
- 2.4 Line 4 - Transitions between HIV Infected States . . . 9
- 2.5 Line 5 - Force of Infection . . . 9
- 2.6 Summary of Model Equations . . . 9

3 Force of Infection **10**
- 3.1 The base rate of infection . . . 10
- 3.2 Determining the partner turnover rates per HIV state . . . 11
  - 3.2.1 Determining the partner turnover rate with specific groups . . . 11
- 3.3 Partnership Turnover Balancing . . . 12
- 3.4 Turnover rate equation flow . . . 13

1 Model Description

The model was written using the Berkeley Madonna modelling software.

For infection of HIV, we consider the following populations:

$$M_s^{j,k}, \qquad F_s^{j,k} \tag{1}$$

denoting the male (M) and female (F) populations by designation according to the subscript state and superscript groups. The subscript denotes the HIV state s. The superscripts denote groups: age group j, and sexual activity group k. To distiguish between sexually active and sexually inactive populations we will use the HIV state $s = 0$ for sexually inactive people. We emphasize that the **superscripts denote the group** while **subscript denotes the state** within a given group.

1.1 Burn In

To allow the demographic composition of the population to reach current empirical age/sex demography, we begin each simulation with a 100-year burn-in. That is, we begin the simulation in 1870, and do not obtain model estimated results until 1970 at the earliest. We introduce HIV infection in the population by spontaneously infecting 0.0001% of sexually active males aged 20-29 in a user defined year between 1970 and 1980.

1.2 Age Group Parameters

The age group, denoted by subscript j, ranges from 0 (the lowest age group) to $J - 1$ the highest age group where there are J total age groups. An age group is defined by the start and end ages of the group. Although age groups may be supplied by the user with any range, our modelling work generally groups ages into 1-year intervals, up to age 70.

1.3 Sexual Activity Group Parameters

Sexual activity group k ranges from 0 to $K - 1$ where there are K total activity groups. Sexual activity is defined by desired sexual partner turnover rate and desired age mixing.

1.4 Age/Sexual Activity Input Parameters

Each doublet (j, k) defines a population (either male or female) existing in age group j, and sexual activity group k. For each such group we allow the following parameters:

1.4.1 Birth rate

We let $b^{(j)}$ be the number of births per unit time per female in age group j. We let b_m and b_f be the fraction of births that are males and females respectively. We enforce the constraint that $b_m + b_f = 1$. The rate of male or female births per unit time per female in a given age

group are then the products of these fractions and the corresponding rate $b^{(j)}$. Finally we assume that the user provides as input vectors that indicates what fraction of all births that end up in sexual activity group k upon reaching sexual debut. We denote these vectors as:

$$b_{m,k} \quad and \quad b_{f,k} \tag{2}$$

where we permit different vectors for males and females. *All of the above birth rate parameters are supplied as user input.*

1.4.2 Background Mortality rate

We let $\mu_m^{(j)}$ and $\mu_f^{(j)}$ denote the background male and female mortality rates (exclusive of HIV related mortality) for age group j. The units of these rates are deaths per unit time per person (as deaths are people these units are just frequency). *These mortality rates are supplied as user input.*

1.4.3 Age of sexual debut and becoming sexually active

We let $\vartheta^{(m,j)}$ and $\vartheta^{(f,j)}$ denote the rate of becoming sexually active per unit time per male or female, respectively, at a specific age j. Then, the earliest potential age of sexual debut, denoted $d^{i,j}$ where i is one of m or f for males or females, can be computed as the minimum j such that $\vartheta^{(i,j)}$ is non-zero. *The parameters $\vartheta^{i,j}$ are input by the user.*

1.4.4 Desired partnership turnover rate

We let $\Delta_s^{(m,j,k)}$ and $\Delta_s^{(f,j,k)}$ be the desired partner turnover rate for age group j and sexual activity group k for males and females respectively in HIV state s. Note that these values are identically zero for $s = 0$, the sexually inactive and HIV uninfected state. These rates are the total number of desired parters of the opposite sex independent of the partner's age group, sexual activity group, or HIV state. *These rates are specified by the user.*

1.4.5 Desired partner mixing parameters

We denote $m_{j'}^{(m,j,k)}$ and $m_{j'}^{(f,j,k)}$ as the percentage of partners that males and females in age group j and activity group k respectively want from age group j' subject to the following constraint:

$$\sum_{j'=0}^{J-1} m_{j'}^{(m,j,k)} = \sum_{j'=0}^{J-1} m_{j'}^{(f,j,k)} = 1 \tag{3}$$

For example, the desired partnership turnover rate can be specified with a target partner age j' such that:

$$\begin{aligned} \Delta_{s,j'}^{(m,j,k)} &= \Delta_s^{(m,j,k)} m_{j'}^{(m,j,k)} \\ \Delta_{s,j'}^{(f,j,k)} &= \Delta_s^{(f,j,k)} m_{j'}^{(f,j,k)} \end{aligned} \tag{4}$$

In general, the mixing matrix is supplied as a generic mixing range and a desired fraction of partners that are older and younger than the age group j. The mixing matrix conforms to

the fact that for certain age groups j, the corresponding age group j' within the mixing range may not contain a sexually active population. In this case, the fractional part of partners in older or younger age groups is adjusted accordingly. This may happen when people at the youngest age at sexual debut desire a fraction of partners to be younger than themselves, or when those at or near the oldest age in the model (supplied by the user but, e.g., age 70) desire a fraction of partners to be older than themselves.

1.4.6 Cross generational mixing

Notice that the age mixing matrix has both gender and sexual activity group dimensions. Age mixing may vary by sexual activity group. In our current work, some women are in sexual activity groups in which they are willing to have much older partners (cross-generational mixing with partners up to 30 years older than themselves). In the current work, this corresponds to sexual activity groups $k = 3$ to $k = 5$, in which women desire the same number of partners as women in groups $k = 0$ to $k = 2$, respectively, but with a different age mixing range. Women in these sexual activity groups desire 100% of their partners to be 1 to 30 years older than themselves. All men in the model may have some cross-generational mixing, but not all women, and the proportion of women who are willing to mix cross-generationally is supplied by the user as a model input.

1.4.7 Sexual Activity migration

We let the quantity $B_{k,k'}^{(m,j)}$ and $B_{k,k'}^{(f,j)}$ denote rates of migrating from sexual activity (Behaviour) group k to sexual activity group k' for age group j for males and females respectively. Though not dictated by the model structure, a common constraint in our work is that sexual activity migration does not directly occur between two non-adjoining sexual activity groups. For example, $B_{1,3}^{(m,j)} = 0$. *These values are supplied by the user.*

1.5 Population States

Having defined a population group (recall this is the superscripts) and all of the parameters related (primarily) to the group, we can now discuss the different **states** that exist in this population (recall that a state is given by the subscript sx). Recall that our populations are denoted by:

$$M_s^{j,k}, \qquad F_s^{j,k} \tag{5}$$

Here we focus on the subscript s which denotes the possible HIV states of the total population (j, k).

1.5.1 HIV state

The subscript s denotes the HIV state of the population. We distinguish between s=0, the HIV uninfected state among sexually inactive groups and s=1, the HIV uninfected state

among sexually active groups. In addition, there are four HIV stages while not on anti-retroviral therapy (ART), the last stage representing AIDS. The four HIV stages are denoted by s=2 to s=5. There are also three HIV states related to HIV infected people on ART, s=6 to s=8. These represent those transiting to ART from HIV stages 1-3 (not yet AIDS), those transiting to ART from HIV stage 4 (AIDS), and those who have been on ART long enough that their HIV-related mortality rate has disappeared so that they only die from background (non HIV-related) mortality.

The rate of transition from one state to another is provided as user input and may include a rate of 0 (for example, the user will not wish to allow those in HIV stage 3, represented by $s = 4$, to transit to HIV stage 2, represented by $s = 3$).

1.6 HIV State Input Parameters

The following input parameters are defined for a given state.

1.6.1 HIV stage and ART transition rates

For any two HIV infected states including those on ART treatment, we let $\gamma_{s,s'}^{(i,j)}$ denote the rate of transition from state s to state s', for gender i and age j. These may equal 0. There are two special cases, both involving HIV uninfected populations. As a reminder, the rate of transition between $s = 0$ (sexually inactive and HIV uninfected) to $s = 1$ (sexually active and HIV uninfected) is determined by $\vartheta^{(m,j)}$ and $\vartheta^{(f,j)}$ for males and females respectively. The second special case involves the rate of transition from $s = 1$ to $s = 2$. This is the force of infection.

1.6.2 HIV/AIDS-related Mortality rate

We let $\mu_s^{(j)}$ denote the HIV stage-dependent mortality. This represents the excess mortality that individuals in HIV stage s incur, over and above background mortality. These mortality rates are independent of gender and sexual activity group. These mortality rates will, by definition, equal 0 for stages $s = 0$ (sexually inactive and HIV uninfected) and $s = 1$ (sexually active but HIV uninfected). The units of these rates are deaths per unit time per person (as deaths are people these units are just frequency). *These mortality rates are supplied as user input.*

1.6.3 HIV infection rate (force of infection)

The force of infection HIV is the heart of the algorithm and will be discussed in a section on its own later. For now, we simply denote the force of infection as $\lambda^{(m,j,k)}$ and $\lambda^{(f,j,k)}$ to denote the force of infection for males and females respectively. We let the force of infection depend on all population group parameters. Before giving the force of infection equations, we now provide the differential equations for the rate of change of population. We will, however,

have as inputs to the model the probability of HIV infection per sex act with an infected partner, which we denote $\phi_{s'}^{(m,j,k)}$ for men and $\phi_{s'}^{(f,j,k)}$ for women. Note that these probabilities depend on the HIV state s' of the partner. We will also supply the number of sex acts per partnership $\psi^{(m,j,k)}$, $\psi^{(f,j,k)}$ which we assume can vary with the index person's gender, age and sexual activity group.

The model was designed such that the user can decide whether to provide probability of HIV transmission per partnership, or per sex act. If the user wishes to base the model on HIV transmission probability per partnership, then the number of sex acts per partnership may be entered as 1, and the probability of transmission per sex act may be used to represent the probability of transmission per partnership.

2 Model Equations

Let I represent either a male or female population. The model equations are then:

$$
\begin{aligned}
\frac{dI_s^{(j,k)}}{dt} &= I_s^{(j-1,k)} - I_s^{(j,k)} - \mu^{(i,j,k)} I_s^{(j,k)} - \mu_s^{(j)} I_s^{(j,k)} + \delta_j \delta_s b_i b_{i,k} \sum_{j'} b^{(j')} F^{(j')} \\
&+ \sum_{k'=0}^{K-1} B_{k',k}^{(i,j)} I_s^{(j,k')} - \sum_{k'=0}^{K-1} B_{k,k'}^{(i,j)} I_s^{(j,k)} \\
&+ \delta_{s-1} \vartheta^{(i,j)} I_s^{(j,k)} - \delta_s \vartheta^{(i,j)} I_s^{(j,k)} \\
&+ \sum_{s'=2, s' \neq s}^{S} \delta_{\mathrm{inf}(s)-1} \gamma_{s',s}^{(i,j)} I_{s'}^{(j,k)} - \sum_{s'=2, s' \neq s}^{S} \delta_{\mathrm{inf}(s)-1} \gamma_{s,s'}^{(i,j)} I_s^{(j,k)} \\
&+ \delta_{\mathrm{sus}(s)-1} \lambda^{(i,j,k)} I_s^{(j,k)}
\end{aligned}
\tag{6}
$$

We will now break down the model equations line-by-line.

2.1 Line 1 - Aging, Births and Deaths

In line one, the first term represents the population aging into the current group. We assume that the population at $j = -1$ is identically zero for any remaining flags/states. The second term is the population leaving the current age group. The third term is the natural mortality rate multiplied by the population in the current group. The fourth term is the HIV/AIDS-related mortality rate multiplied by the population in the current group and current HIV state.

The last term is the birth term. Here we are using the notation that $\delta_p = 1$ if and only if $p = 0$ and is zero otherwise. Therefore the product of the four delta functions indicates that this term will only contribute to age group zero, and the sexually inactive population state $s = 0$. The summation gives the total births due to all age groups. Note that we are abusing notation: $F^{(j)}$ without subscripts indicates that all HIV states are to be summed over as well. This sum is then multiplied by the fraction $b_{i,k}$ which gives the fraction of the gender i birth rate that contributes to sexual activity group k, and b_i which gives the fraction of the birth rate that contributes to age group i. Remember that before sexual debut, the partner turnover rate in all sexual activity groups = 0.

2.2 Line 2 - Sexual Activity Group Migration

Line 2 represents the migration terms between activity groups. Recall that sexual activity is defined by sexual partner turnover rate, and age mixing preference. The first term accounts for the total population arriving in behaviour group k from all other groups k'. The second term accounts for the total population leaving group k for other groups k'.

2.3 Line 3 - Sexual Activity Debut

Line 3 represents the population change rates due to the current group becoming sexually active. It is assumed that the rates $\vartheta^{(i,j)}$ are zero below the earliest potential age of sexual debut. The first term adds to the $s = 1$ state (HIV uninfected but sexually active) from the inactive state $s = 0$. This same number is then removed from the $s = 0$ state in the second term.

2.4 Line 4 - Transitions between HIV Infected States

In this line we have the transitions between HIV infected states, including those states that include ART treatment. Here we use the function inf(s).

inf(s) = 1 if s >1, 0 otherwise

That is, inf(s) returns 1 provided state s is one of the HIV infected states; that is, if $s \neq 0$ and $s \neq 1$. Recall that, as always, $d_p = 1$ if and only if $p = 0$ and is zero otherwise. Therefore the first term adds to the current infected state while the second term subtracts from other infected states.

2.5 Line 5 - Force of Infection

This moves us from state $s = 1$ (HIV uninfected but sexually active) to state $s = 2$ (HIV infected stage 1).

2.6 Summary of Model Equations

Every term in the above equation is known (supplied by the user) except for the force of infection. This must be computed and we give the equations in the next section.

3 Force of Infection

In order to compute the force of infection we recall the following user input parameters:

- $\Delta_s^{(m,j,k)}$,$\Delta_s^{(f,j,k)}$ the desired partner turnover rates.
- $m_{j'}^{(m,j,k)}$, $m_{j'}^{(f,j,k)}$ the mixing relations i.e. the proportion of the total desired partners belonging to age group j'.
- $\phi_{s'}^{(m,j,k)}$,$\phi_{s'}^{(f,j,k)}$ the transmission probability of HIV per sex act. Although the model allows this probability to vary by all groups and states, in most of our work we will not vary this parameter by age. In some cases, we may vary this probability by sexual activity group, to be used as an indirect proxy for a higher likelihood of co-infection with other STDs among those in the higher sexual activity groups.
- $\psi^{(m,j,k)}$, $\psi^{(f,j,k)}$ the number of sex acts per partnership.

3.1 The base rate of infection

The base rate of infection of HIV **per partnership** can be computed using the probability of infection per sex act and the number of sex acts per partnership. The probability of not being infected in a single sex act is $(1-\phi_{s'}^{(i,j,k)})$ where i is either m or f for males or females. Then the probability of being infected in the total number of sex acts (assuming the partner is infected) is:

$$\beta_{s'}^{(i,j,k)} = 1 - (1-\phi_{s'}^{(i,j,k)})^{\psi^{(i,j,k)}} \tag{7}$$

Here $\beta_{s'}^{(i,j,k)}$ represents the probability of getting infected per partner in HIV state s'. In our current modelling work, we fit our model using the probability of transmission per partnership as input, rather than the probability of transmission per sex act. We therefore simply set ψ to 1 for all groups and states, and consider ϕ as the probability of transmission per partnership. Therefore:

$$\beta_{s'}^{(i,j,k)} = 1 - (1-\phi_{s'}^{(i,j,k)}) = \phi_{s'}^{(i,j,k)} \tag{8}$$

The probability of infection from any partners in HIV state s' is then equal to one minus the probability of not being infected by any partner in state s':

$$\lambda_{s'}^{(i,j,k)} = 1 - (1-\beta_{s'}^{(i,j,k)})^{\Delta_{s,s'}^{(i,j,k)}} \tag{9}$$

Here, $\Delta_{s,s'}^{(i,j,k)}$ is the partner turnover rate with people infected and in HIV state s', which we will see later is derived from $\Delta_s^{(i,j,k)}$, the total desired partner turnover rate entered as input to the model. That is, $\Delta_s^{(i,j,k)}$ is the sum:

$$\Delta_s^{(i,j,k)} = \sum_{s'} \Delta_{s,s'}^{(i,j,k)} \tag{10}$$

where $\Delta_{s,s'}^{(i,j,k)}$ is the product of $\Delta_s^{(i,j,k)}$ and the proportion of the partnership pool with whom group (i,j,k) mixes, that is in HIV state s'.

The force of infection is then equal to one minus the probability of not being infected by any partner in any HIV state:

$$\lambda^{(i,j,k)} = 1 - \prod_{s'} (1 - \lambda_{s'}^{(i,j,k)}) \tag{11}$$

3.2 Determining the partner turnover rates per HIV state

To compute the force of infection we must compute $\Delta_{s,s'}^{(i,j,k)}$, the desired partner turnover rate with people in a given state. The number of available partners in a given group and state is:

$$P_s^{(i,j,k)} = I_s^{(j,k)} \Delta_s^{(i,j,k)}, \quad s > 0, \quad 0 \text{ otherwise} \tag{12}$$

Recall that HIV state 0 is used to represent the sexually inactive population and uninfected with HIV. It is assumed that the desired turnover rate for the sexually inactive state $s = 0$ is identically zero. The partner turnover rate with a given state s' is:

$$\Delta_{s,s'}^{(i,j,k)} = \sum_{j'=0}^{J-1} \sum_{k'=0}^{K-1} \Delta_s^{(i,j,k),(j',k')} \frac{P_{s'}^{(i',j',k')}}{\sum_{s'} P_{s'}^{(i',j',k')}} \tag{13}$$

This equation is explained as follows. Denote the opposite gender as i'. The last term provides the ratio of partnerships on offer for a given state s' over all to the total available partners on offer in all HIV states. The newly introduced term $\Delta_s^{(i,j,k),(j',k')}$ is the number of partnerships desired with the primed group (yet to be computed). That is, it is a subset of $\Delta_s^{(i,j,k)}$: $\quad \Delta_s^{(i,j,k),(j',k')} \subset \Delta_s^{(i,j,k)}$. Summing this over all age and sexual activity groups gives the total number of desired partners with a given state s'.

3.2.1 Determining the partner turnover rate with specific groups

We now determine $\Delta_s^{(i,j,k),(j',k')}$. This is computed based on age mixing preferences and sexual activity group mixing preferences.

Specifically, $\Delta_s^{(i,j,k),(j',k')}$ is computed as follows:

$$\Delta_s^{(i,j,k),(j',k')} = \Delta_s^{(i,j,k)} \left[(1 - \epsilon_k)\delta_{k,k'} + \epsilon_k \left(\frac{\sum_{s'} P_{s'}^{(i',j',k')}}{\sum_{k''=0}^{K-1} \sum_{s'} P_{s'}^{(i',j',k'')}} \right) \right] m_{j'}^{(i,j,k)} \tag{14}$$

The first multiplicative term in the above equation is the overall desired turnover rate for the population with HIV state s, gender i, age group j, and sexual activity group k, provided as user input. Next we have the **activity group assortativity parameter** ϵ_k which ranges between 0 and 1. In the case that ϵ_k is zero, the first large multiplicative term tells us

that there is no cross-mixing by sexual activity group; this would represent 100% assortative mixing by activity group. In the case that $\epsilon_k = 1$, the first term is zero and the first multiplication is by the ratio of the total partnerships on offer in group k' to the total partnerships on offer over all activity groups k''. Therefore multiplying this ratio by the desired partnership turnover rate $\Delta_s^{(i,j,k)}$ would distribute these partnerships according to the available ratio of partnerships on offer from the partner group. This would represent 100% random mixing by activity group, based on partnerships available. Any value of ϵ_k greater than 0 and less than 1 would represent partially assortative and partially random mixing.

We then multiply by $m_{j'}^{(i,j,k)}$, that is the mixing factor with age group j' in order to adjust the desired partnerships by the fraction of desired partners in age group j'.

3.3 Partnership Turnover Balancing

Most often, the total number of partnerships requested from j', k' for j, k will not match exactly the total number of partnerships requested from j, k for j', k'. That is, most often:

$$\sum_{s=1}^{S} I_s^{(j,k)} \Delta_s^{(i,j,k),(j',k')} \neq \sum_{s'=1}^{S} I_{s'}^{\prime (j',k')} \Delta_{s'}^{(i',j',k'),(j,k)} \tag{15}$$

We now discuss balancing partner turnover rates to match partnerships available. This is done as follows:

$$BL^{(j,k),(j',k')} = \frac{\sum\limits_{s=1} F_s^{(j,k)} \Delta_s^{(f,j,k),(j',k')}}{\sum\limits_{s=1} M_s^{(j',k')} \Delta_s^{(m,j',k'),(j,k)}} \tag{16}$$

This equation is explained as follows: The sum of F in the numerator over s gives the total active female population (note that $s = 0$ is not included in the sum). Multiplying this population by by the rate $\Delta_s^{(f,j,k),(j',k')}$ gives the total number of partners that this population desires with men in the primed group.

The denominator is analogous except for men and gives the total number of partners that men in the primed group want with women in the unprimed group.

If the ratio of these two quantities is more than unity, women in the index (unprimed) group desire more partners per time than men in the target (primed) group. If the ratio is less than unity, men in in the target group desire more partners than women in the index group.

We then adjust the partner turnover rates as follows:

$$\begin{aligned} \Delta A_s^{(m,j,k),(j',k')} &= \Delta_s^{(m,j,k),(j',k')} \times \left(BL^{(j,k),(j',k')}\right)^{\theta} \\ \Delta A_s^{(f,j,k),(j',k')} &= \Delta_s^{(f,j,k),(j',k')} \times \left(BL^{(j,k),(j',k')}\right)^{-(1-\theta)} \end{aligned} \tag{17}$$

Where θ is a compromise parameter between 0 and 1. If θ is zero, men get what they want, i.e. they get their desired rates. In this case, if BL is greater than unity (women desire more than men), the rate for women will be reduced by a ratio of BL. If θ is 1, women get what they want. If BL is greater than 1, this means that the rate for men will be increased by a factor of BL.

3.4 Turnover rate equation flow

Once the adjusted partner turnover rates are computed in eq (17), these adjusted rates are used to compute the turnover rates with a given state s in eq (13). From the state turnover rates we compute the HIV infected turnover rates in eq (10) and finally the force of infection in eq (11).
