# Supplementary material for: The dual impact of antiretroviral therapy and sexual behaviour changes on HIV epidemiologic trends in Uganda: a modelling study
Source: Sex Transm Infect. 2014 Feb 24;90(5):423–9. doi: 10.1136/sextrans-2013-051219 (PMC4112492; doi:10.1136/sextrans-2013-051219)
Supplement: Web table [file sextrans-2013-051219-s2.pdf]

Table S1. Model Input Ranges (note: in all subscripts, i=gender, j=age, k=sexual activity group)

**Model Input Parameter** (Symbol used in equations)	**Range**	**Source**	**Value in top fitting scenario**
**Demography**			
Fertility rates per year (b^j)	Fixed (non-varying)	Analysis of Masaka (Uganda) cohort data	Age 15-19: 0.180<br>35-39: 0.211<br>20-29: 0.333<br>40-44: 0.079<br>30-34: 0.275<br>45-49: 0.028
Non HIV-related mortality rates per year (μ_i^j)	Fixed (non-varying):	Cohort data analysis, fitted to Brass life table model[1]	Life expectancy<br>Females: 69 yrs<br>Males: 66 yrs
**Biology**			
HIV-related mortality rate (μ_s), where s=one of the HIV (not yet AID) HIV states	0.04-0.10/year	Cohort anlysis	0.065/year
AIDS-related mortality rate (μ_s), where s = AIDS state	0.7-1.3/year (~ 0.04-0.11/month)	[2]	1.29/year
HIV infection probability per partnership (ϕ_s^i)<br><br>Infection probabilities for HIV stage 1, 3, 4 are multiples of Stage 2, to ensure infectiousness from highest to lowest in stages 1, 4, 3, 2, respectively. (Stage 4 = AIDS)	HIV Stage 2:<br>M to F: 0.01-0.08<br>F to M: (male prob) * (0.05-0.90)<br><br>M to F by stage:<br>Stage 1: 0.05-0.72<br>Stage 3: 0.0125-0.54<br>Stage 4: 0.0188-0.71	[3-5]	M->F: 6.7%<br>F->M: 5.5%<br><br>M->F: S1: 29%<br>S3: 19%<br>S4: 28%<br>F->M: S1: 21%<br>S3: 14%<br>S4: 21%
Mean duration in each HIV stage = 1/(rate of movement between HIV state s and s')<br>($\gamma_{s,s'}$)	Fixed (non-varying)	[2, 6]	Stage 1: 5 months,<br>stage 2: 7 years,<br>stage 3: 1.5 years,<br>stage 4: 10 months
Year of first HIV infection	1970-1980	Earlier than 1982 [7]	1976
**Sexual Behavior**			
Earliest potential age at sexual debut	11-15	Cohort data analysis	14
Rate of becoming sexually active ($\vartheta^{i,j}$)	Beginning with earliest potential age at sexual debut, the rate of becoming sexually active first increases with age, and then decreases.<br><br>Our data show that those not sexually active by a certain age have a very low rate of becoming sexually active.	Cohort data analysis	Age / Sex / Rate/year<br>14-15 M 0.032<br>F 0. 023<br>16-19 M 0.128<br>F 0.138<br>20-24 M 0.214<br>F 0.253<br>25-28 M 0.160<br>F 0.184<br>29+ M 0.096<br>F 0.161
Desired partner turnover rate by gender, age, sexual activity group, not in AIDS state ($\Delta^{i,j,k}$)	Until age 30, the ranges for ($\Delta_{i,j,k}$):<br>High activity: 20-90<br>Middle activity: 1-5	Cohort data analysis	$\Delta_{i,j,k}$ for Age < 30:<br><br>Sex Group Rate/yr

	Low activity: (middle activity rate) * (0.1 – 0.5)<br><br>Age 30+: $\Delta_{i,j,k}$ reduces by 3%/year		M High 68.24<br>Mid 1.99<br>Low 0.25<br>F High 36.62<br>Mid 2.96<br>Low 1.15
Desired partner turnover rate by gender, age, sexual activity group, in AIDS state ($\Delta^{i,j,k}$)	($\Delta^{i,j,k}$) * (0.20 – 0.80)	Cohort data analysis (with limited data)	0.41
Proportion entering each activity group at sexual debut - based on partner turnover desire (b_k)	High: 0.1%-3.0%<br>Middle: 30% - 85%<br>Low: 100%-high-middle	Cohort data analysis	High: 2.7%<br>Middle: 62.0%<br>Low: 35.3%
Cross generational age mixing: Proportion entering each 'cross generational' activity group – based on desired age mixing:<br><br>(b_k) * (proportion of women willing to have cross generational partners)	Range at sexual debut: 0-50%<br><br>Rate of leaving cross-generational age mixing group: 0.00-0.10	Cohort data analysis	31% at sexual debut.<br><br>Starting at age 25, the rate of leaving this compartment (among women) = 0.060/year.
Age mixing (non-x-generational)<br><br>Applies to partnerships that are not with women willing to engage in cross-generational sex.	Maximum age difference between partners: 2-7 years.<br><br>80% of partnerships are desired with older males, while 20% with younger/same age males.	Cohort data analysis	± 3 years
Assortativity of mixing by sexual activity group (ε_k)	0 to 1<br><br>0 = assortative<br>1 = proportional to partnerships on offer	[8]	0.66
Balancing parameter (θ)	0 to 1<br><br>0 = men get what they are requesting and women increase/decrease partnerships to match male demand.<br>1 = women get what they request.<br>Between 0-1 = a compromise.	[9]	0.98
Year of intentional behavior change	1st year of change: 1988-1991<br>2nd year of change: 1998-2000	Cohort data analysis, and [10]	1988.7<br>1998.0
Factor changing desired sexual partner turnover rates:<br>New $\Delta_{i,j,k}$ = (Old $\Delta_{i,j,k}$)*factor	1988-1991: 0.44 – 0.99<br>1998-2000: 1.01 – 2.01	Turnover rates change gradually over 2 years.	1988.7: 0.97<br>1998.0: 1.02

References

1. Brass, W., *Methods for Estimating Fertility and Mortality from Limited and Defective Data.* An Occasional Publication, 1975(Chapel Hill: POPLAB).
2. Morgan, D., et al., *HIV-1 infection in rural Africa: is there a difference in median time to AIDS and survival compared with that in industrialized countries?* Aids, 2002. **16**(4): p. 597-603.
3. Wawer, M., et al., *Rates of HIV-1 Transmission per Coital Act, by Stage of HIV-1 Infection, in Rakai, Uganda.* J Inf Dis, 2005. **191**: p. 1403-1409.
4. Powers, K., et al., *Rethinking the heterosexual infectivity of HIV-1: a systematic review and meta-analysis.* Lancet Infect Dis, 2008. **8**: p. 553-63.
5. Rottingen, J. and G. Garnett, *The Epidemiological and Control Implications of HIV Transmission Probabilities Within Partnerships.* Sex Transm Dis, 2002. **29**(12): p. 818-827.
6. Todd, J., et al., *Time from HIV seroconversion to death: a collaborative analysis of eight studies in six low and middle-income countries before highly active antiretroviral therapy.* AIDS, 2007. **21**(suppl 6): p. s55-s63.
7. Serwadda, D., et al., *Slim disease: a new disease in Uganda and its associations with HTLV III infection.* The Lancet, 1985. **2**: p. 849-852.
8. Hallett, T.B., et al., *Declines in HIV prevalence can be associated with changing sexual behaviour in Uganda, urban Kenya, Zimbabwe, and urban Haiti.* Sex Transm Infect, 2006. **82 Suppl 1**: p. i1-8.
9. Garnett, G. and R. Anderson, *Balancing sexual partnerships in an age and activity stratified model of HIV transmission in heterosexual populations.* J Math Applied in Med & Biology, 1994. **11**: p. 161-192.
10. Kamali, A., et al., *Seven-year trends in HIV-1 infection rates, and changes in sexual behaviour, among adults in rural Uganda.* AIDS, 2000. **14**(4): p. 427.
